# An Investigation of the Constitution of the Mercury-Tin System^1^

**DOI:** 10.6028/jres.067A.008

**Published:** 1963-02-01

**Authors:** Duane F. Taylor, Claire L. Burns

## Abstract

An investigation of the constitution of the mercury-tin system was made by a combination of three techniques: differential thermal analysis; diffusion and chemical analysis; and X-ray diffraction. The mercury-silver-tin system is of interest because it is the basis of dental amalgam, the most important single dental restorative material. Information as to the constitution of these alloys is incomplete for both the ternary system and the mercury-tin binary system. This study was devoted to the investigation of the mercury-tin system as a prerequisite to a study of the ternary alloys. The results obtained by the various methods are not in complete agreement. They indicate that the system is more complex than previously reported. Additional evidence for the beta phase as reported by Prytherich was found but the composition limits and eutectoid temperature remain to be confirmed. The gamma phase composition limits were found to differ from earlier values. Corroborative data for Gayler’s delta phase and possible evidence for a previously unreported epsilon phase have been found by X-ray diffraction. The thermal analysis results indicate the possible existence of additional phases unconfirmed by other methods. A modified mercury-tin phase diagram based upon these findings is proposed.

## 1. Introduction

Dental amalgam has been developed to a stage where it is in many ways the best restorative material available to the dentist, and is used in more than three-fourths of all dental fillings. Nevertheless, it has certain undesirable properties which limit its usefulness, such as its tendency to flow under low compressive loads, and its susceptibility to brittle fracture at moderately high loading rates. The development of these alloys to date has been largely by empirical methods. Hope for further improvement appears to depend upon a better knowledge of the underlying metallurgy.

The mercury-silver-tin system, on which dental amalgams are based, has been studied by several workers since Joyner (1)^2^ published the results of the first investigation of the system in 1911. However, progress in developing an understanding of the ternary alloys has been impeded by the lack of a well established diagram for the mercury-tin binary system. Portions of the diagram are incomplete and much of the remainder is in dispute. This study was undertaken with the purpose of improving the knowledge of the mercury-tin alloys as an essential first step toward the understanding of dental amalgams.

## 2. Previous Work

The number of published investigations of the mercury-tin system is small and many of them are confined to studies of portions of the system. The experimental difficulties occasioned by the low melting point of mercury, and the lack of interest caused by the limited commercial application of these materials, have combined to restrict the amount of effort devoted to the study of these alloys.

**Figure 1:**
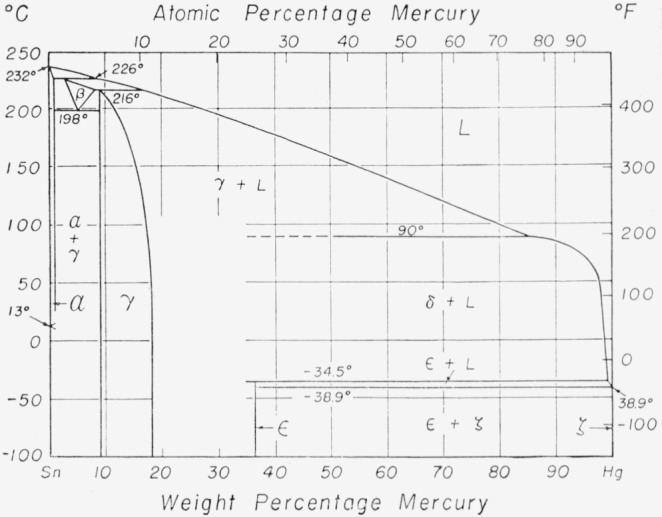
The mercury-tin constitution diagram [2].

Figure 1 shows the currently accepted diagram for the mercury-tin system as given in the Metals Handbook [2]. It differs only in minor detail from that given by Hansen [3]. The liquidus is well established, having been investigated by several authors [4, 5, 6, 7]. Of these the work of van Heteren [6] was the most extensive and probably the most precise, but there is good agreement between his results and those of the others.

The solubility limit of mercury was determined from the electrode potential measurements of van Heteren [6] and the X-ray diffraction studies of Stenbeck [8]. The gamma phase was first identified by von Simson [9], who established the composition limits essentially as shown in figure 1. Stenbeck [8] confirmed her findings and reported evidence of an additional structure, presumably of higher mercury content.

The beta phase was discovered by Prytherch [10], whose work unfortunately has never been reported except to the extent that it was quoted by Gayler [11]. It appears that Prytherch’s [10] diagram was based primarily upon thermal analysis data, showing an arrest at the beta peritectic temperature. The existence of the beta phase was confirmed by the high temperature X-ray diffraction studies of Raynor and Lee [12], although this finding is in apparent conflict with that of Schubert et al. [13], who concluded that the beta and gamma phases were identical.

The existence of a delta phase has been a matter of some dispute. Gayler [11] obtained a series of arrests in the course of thermal analysis of high mercury alloys, which she attributed to a proposed delta phase. Her observations on ternary alloys also appear to require the existence of such a phase. Troiano [14] also supported the existence of a delta phase, but his X-ray findings have been contested by Wainwright [15]. More recent work by Ryge, Moffett, and Barkow [16], Fairhurst and Ryge [17] and Dreiner [18] has produced no evidence for the existence of the delta phase. The uncertainty about the delta phase is indicated by the blank region in figure 1 where this phase would be expected to appear and by the inclusion of portions of the delta peritectic line.

The evidence for the epsilon phase as shown in the figure is almost equally weak. The indicated peritectic temperature is based upon van Heteren’s [6] work, and appears well established, but the composition is dependent upon Prytherch’s [10] unpublished findings.

The lack of agreement between the results obtained by different methods, and between those of different authors employing the same or similar methods, has caused any conclusions about the high mercury solid phases to be very speculative. This uncertainty is carried over to the silver-tin-mercury ternary diagram where the tin-rich corner is largely unknown.

## 3. Choice of Experimental Methods

From a number of possible methods of study, three were selected that appeared to be particularly well suited to the alloy system and to complement each other. These methods were differential thermal analysis, diffusion and chemical analysis, and X-ray diffraction. These methods had the added advantage that they would be similarly useful in an extension of the work to ternary alloys.

Differential thermal analysis, a traditional and basic approach, has several specific advantages. In the mercury-tin system, thermal analysis was the method used in the original detection of both the beta and delta phases. Its employment in this study offered a direct check on those findings. At the same time, it provided a tie-in to the well established liquidus data. The main drawback to this method is the susceptibility to suppression of certain phases in peritectic systems at heating and cooling rates normally employed. This tendency can be partially offset by the use of high-sensitivity differential techniques and low heating and cooling rates.

Diffusion and chemical analysis was chosen as a second method primarily because the diffusion can be perform ed iso thermally and the prolonged retention of nonequilibrium phases formed at higher temperatures can be avoided. It also parallels the normal procedures in the use of dental amalgams and thus might shed some additional light upon the mechanisms of the amalgam setting reaction. The method is more effective in the determination of composition limits than in the determination of the range of temperature stability.

X-ray diffraction was selected as an adjunctive method to the thermal analysis and diffusion techniques. The ability of X-ray diffraction to identify individual crystal structures and thus demonstrate the presence of an individual phase in a mixture is of particular importance when used with diffusion specimens. As a separate method of identification it permits the confirmation or refutation of the phase sequence proposed by chemical analysis.

## 4. Materials Used

The compositions of the mercury and tin used in this study are given in tables 1 to 3. The mercury used was obtained from the Inorganic Chemistry Section of the National Bureau of Standards, where it was refined. The values of table 1 are maximum values from repeated analyses of various lots, rather than that for the particular lot used.

Two lots of tin were used. One lot consisted of Baker and Adamson Reagent Grade Tin Sticks produced by the General Chemical Division of Allied Chemical and Dye Corporation. This metal was used for a limited number of the early tin diffusion specimens. The manufacturer’s reported analysis is given in table 2. The other lot of tin, which was used for all remaining diffusion specimens as well as all thermal analysis and X-ray diffraction specimens, was Tadanac Brand Tin Shot obtained from the Consolidated Mining and Smelting Company of Canada Limited. The spectroanalysis of this tin, table 3, was performed by the Spectrochemistry Section of the National Bureau of Standards.

## 5. Differential Thermal Analysis

### 5.1. Equipment

The results of previous studies of the mercury-tin system that employed thermal analysis as a technique [5, 6, 10, 11] led to the expectation that the thermal effects of interest were apt to be small. In addition, the desirability of using low heating and cooling rates to permit closer approaches to equilibrium was expected to increase the problem of observing small heat effects. In order to obtain sufficient sensitivity, a differential method was employed using mercury as a reference substance. This method produces significant increase in sensitivity and is well suited to the detection of transformation in solid alloys [19].

The furnace used for this work was a vertical tube furnace, 11 inches in diameter and 21 inches in length with a lumen 1⅛ inches in diameter. The power supply to the furnace was provided by three transformers arranged in series. The first was a constant voltage transformer which served to suppress fluctuations in line voltage. The second and third transformers were variable transformers used to provide sensitive control of furnace temperature.

**Figure 2:**
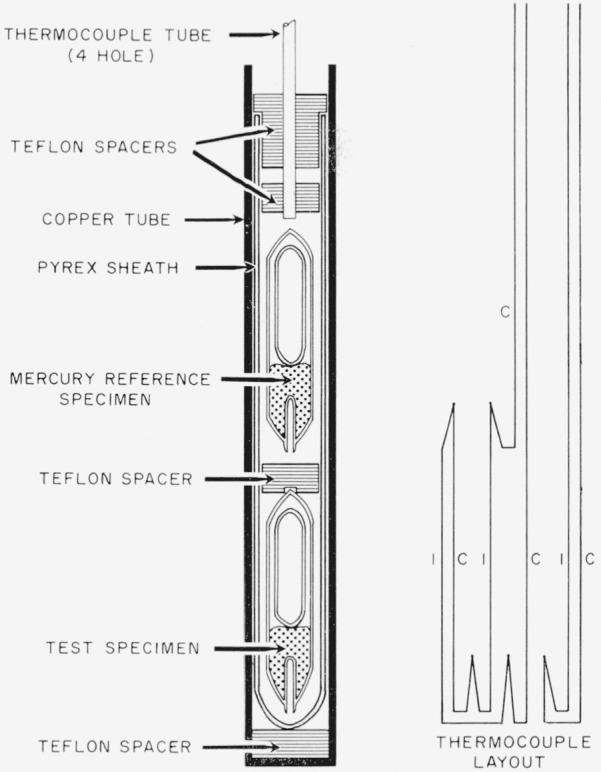
Thermal analysis specimen arrangement.

The specimens were assembled for insertion in the furnace as shown in figure 2. The temperature- indicating and differential thermocouples were inserted in the specimens, and both the experimental and reference specimens were placed in a Pyrex sheath. A Teflon spacer separating the two specimens was drilled and grooved to permit passage of the thermocouples, A similar spacer above the reference specimen served to hold the end of the porcelain thermocouple tube. This tube also passed through the flanged Teflon plug which closed the upper end of the sheath.

The assembly in turn fitted inside a heavy-wall copper tube which served to minimize the temperature gradient along the furnace. The flange on the Teflon plug substantially filled the inside diameter of the copper tube. When the copper tube and its contents were placed in the furnace, a plug rolled from sheet asbestos was placed around the thermocouple tube and slid down until it filled the furnace lumen just above the top edge of the copper. A flanged magnesite plug was then added to close the upper end of the furnace and to serve simultaneously as a support for the thermocouple tube. Under normal circumstances the copper tube was not removed from the furnace when the specimen was changed but was allowed to remain in the furnace as a liner.

Figure 2 also shows a schematic drawing of the thermocouple arrangement. A 28-gage iron-constantan couple was used to measure the temperature of the specimen. The hot junction of this couple was located in the thermocouple well of the test specimen.

The temperature differential was measured by means of a two-junction iron-constantan thermopile arranged as shown in figure 2.

### 5.2. Specimen Preparation

The thermal analysis specimens were prepared in Pyrex tubes with reentrant thermocouple wells, similar in design to those employed by Murphy [20]. In order to maintain approximately constant areas for heat transfer, the specimens were prepared to constant volume rather than constant weight. The volume used was 3 ml, which produced a specimen about 30 mm long with the end of the thermocouple well approximately centered in the specimen. Weighed amounts of tin and mercury were placed in the tube and a sealed stuffer tube was added to fill most of the space below the intended seal. The tube was then repeatedly evacuated and flushed with dry hydrogen and was finally sealed with a residual hydrogen pressure of 2 to 5 mm of mercury. The alloys were then homogenized by heating to 250 °C and holding at that temperature for at least 1 hour, with repeated vigorous shaking. The tubes were then quenched in water at 20 to 25 °C and placed in an air bath at the selected annealing temperature.

The nominal composition of the mercury-tin alloys prepared for thermal analysis is given in table 4. The compositions are given in both weight and atomic percent; however, for convenience of discussion the specimens will normally be referred to only in terms of their composition in weight percent. A limited number of analyses indicated that the actual composition of specimens prepared by this technique did not differ significantly from the nominal values.

### 5.3. Experimental Procedure

The individual specimen was removed from the annealing oven and assembled with a reference specimen of pure mercury, as shown in figure 2. No attempt was made to maintain the specimen at the annealing temperature during this process, although the operation was completed as rapidly as possible. In the case of specimens annealed at the higher temperatures (such as 85 °C) the necessity of manipulating the specimens resulted in their being cooled at least to a temperature where they could be readily handled. After the thermocouples had been inserted and both specimens positioned in the outer glass tube, the assembly was inserted into the furnace. The furnace temperature had been adjusted previously to an initial temperature at or below the annealing temperature so that the first test run in each instance was a heating run.

Most of the individual heating and cooling runs were made at a constant applied voltage. This resulted in a high initial heating or cooling rate as the temperature distribution within the furnace adjusted to the changed power input. After a transient period the rate was found to stabilize and almost any rate desired in the 200 °C range of interest could be obtained by the proper selection of the applied voltage. In a limited number of instances, where very slow rates seemed desirable, a clock drive was employed to vary the voltage.

Readings of the specimen temperature and of the differential temperature were made at regular intervals, normally every 2 min, except that at the higher heating and cooling rates 1-min readings were taken. In addition, an attempt was made to obtain extra readings at the maximum and minimum differential readings. With the heating rates most commonly employed, this procedure led to readings in intervals varying from 0.2 to 0.5 °C.

After the initial heating run and all subsequent heating and cooling runs, the specimen was held at a constant temperature for a period of time to permit the temperature distribution in the furnace to stabilize and to promote at least partial equilibrium of the specimen. In some cases in which substantial variation occurred between the results of annealed and nonannealed runs with the same specimen, it was returned to the oven for extended reannealing prior to additional tests.

### 5.4. Results

A total of 153 heating and cooling curves were run on the 18 experimental alloys and the pure mercury and pure tin calibration samples. A minimum of six test runs was made on each composition. Heating and cooling runs were customarily alternated with varying annealing times preceding each heating curve. The results are divided into three groups for convenience of discussion: Liquidus determinations, low mercury alloys, and high mercury alloys.

#### a. Liquidus Determination

The liquidus temperature was determined for each composition studied by both heating and cooling curves. Table 4 presents the observed liquidus temperatures for the alloys studied. Since the liquidus was already reasonably well established, a change in technique to reduce the uncertainty did not appear justified. The observed values are in good general agreement with earlier values, though averaging slightly lower than those of van Heteren [6].

#### b. Low Mercury Alloys 0 to 18 Percent Mercury

The results from alloys containing 0 to 18 percent mercury are conveniently considered as a group. The alloys cover the alpha, beta, and gamma regions of the diagram (see fig. 1) and were chosen to study the relationship of those phases. Table 5 lists the temperature of each arrest found and the estimated uncertainty of the determination. It also indicates the composition range of the specimens for which the arrest was detected and, where possible, identifies the associated phases with the type of reaction causing the arrest.

The results on the 0–18 percent mercury group of specimens appear to confirm most of Prytherch’s [10] diagram for this composition range. Definite arrests were obtained at 223.0 ±0.5 °C and 213.9 ±0.5 °C. These values correspond closely to his peritectic temperature for the beta and gamma phases. Supercooling was a consistent problem in the cooling curves, particularly for the specimens containing 10 percent or less of mercury. The attainment of equilibrium in annealed samples prior to determining heating curves was also very difficult. Indeed some of the heating curves were more readily rationalized by an assumption of complete nonequilibrium conditions, that is, no interaction between phases. These observations are probably best substantiated by reference to an example.

**Figure 3:**
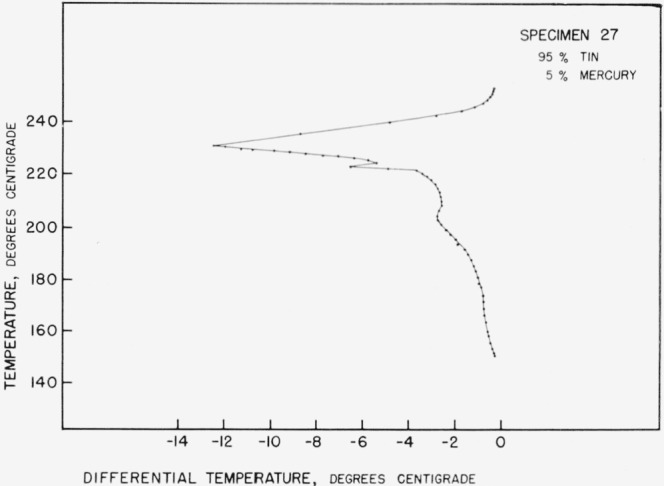
Heating curve for 5% *Hg* 95% *Sn* alloy.

Figure 3 presents the results of a heating run on specimen 27 (5% Hg, 95% Sn). The specimen had been annealed at 85 °C for 71 days prior to this test. This specimen, as well as the 0, 2, and 7 percent mercury specimens, showed no thermal effects below 150 °C on any heating or cooling run.

The main peak at 232.0 °C obviously coincides with the melting point of pure tin and the portion of the curve between there and 225 °C with the equilibrium between alpha and liquid. Similarly the very sharp peak at 222 to 223 °C represents the peritectic decomposition of the beta phase. The identification of the remainder of the curve becomes increasingly difficult as lower and lower temperatures are considered. It does not appear possible to reconcile these portions of the curve with figure 1.

#### c. High Mercury Alloys, 18 to 80 Percent Mercury

As a group, these alloys produced a surprising number and variety of thermal effects. Some of these were strong, routinely detected arrests, while others were much weaker and appeared much less consistently. Of these arrests, some are definitely associated with phase changes, but others may be artifacts or due to second order effects such as superlattice formation or even specific heat anomalies in a single phase. The stronger the arrest and the more often it was obtained, the more precisely can its temperature be determined.

### 5.5. Discussion

The results obtained on thermal analysis of the samples containing from 18 to 80 percent mercury indicate the occurrence of an unusually large number of arrests. To explain all of the observed arrests as phase changes would require an extremely complicated diagram, particularly when it is recognized that all of the required phases must almost certainly contain less than 30 percent mercury.

Of the arrests observed, four seem most likely to be identified with phase transformations, those at 118.0, 106.1, 91.4, and 67.1 °C. Each of these arrests are relatively strong, appear in both heating and cooling curves and in specimens over a considerable concentration range. In at least one instance each, these arrests have appeared as sharp discontinuities of the type normally associated with peritectic decompositions. The remaining arrests are deficient in one or more of these qualifications.

Cooling curve arrests are most pronounced when the phase of interest is the first or second formed from the liquid on cooling. Because of the shape of the mercury-tin liquidus this condition is met for this phase only in alloys of very high mercury content where the total amount of solid formed is small and the latent heat is thus reduced. Annealing to equilibrium just above the anticipated temperature of the arrest is the preferred method of procedure, but the annealing time required for the last of a series of peritectic phases can be very long.

**Figure 4:**
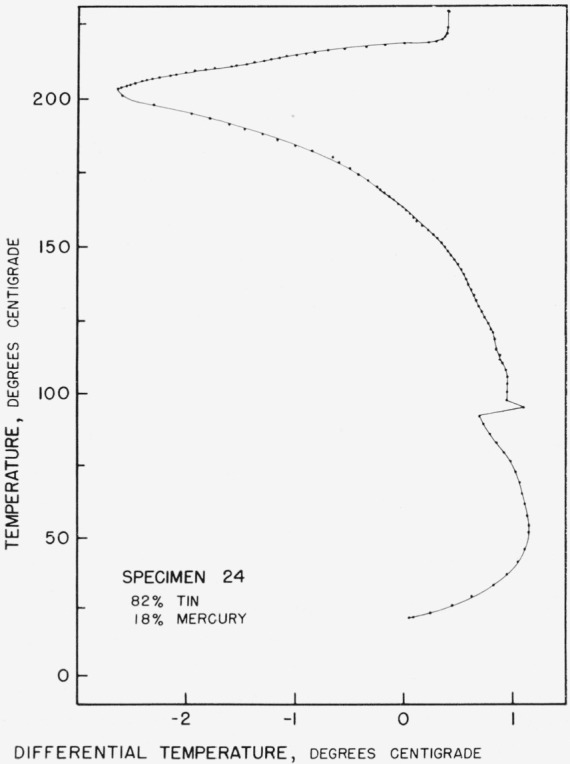
Heating curve for 18% *Hg* 82% *Sn* alloy.

The evidence for the arrest at 203.5 °C is based on heating curve evidence of the sort seen in figure 4. It has failed to appear in any of the cooling curves where it would be expected if it represents a peritectic temperature. It appears in many instances that when annealing conditions have been such as to produce a coarse structure, the phases formed during low temperature annealing tend to persist to their melting points, with little evidence of interaction below that point. The arrest at 203.5 °C is believed to be an artifact of this type.

The arrests observed in low mercury alloys appear to confirm the diagram for the high temperature regions as proposed by Prytherch [10], although the temperatures themselves are in better agreement with those of Hansen [3]. The results of the beta eutectoid temperature determinations may serve as confirmation of the reported value, but are probably inadequate as an independent determination. The liquidus values are in general agreement with earlier results.

## 6. Diffusion and Chemical Analysis

The use of diffusion and chemical analysis as an experimental method for the study of tin amalgams offers several advantages. This experimental technique avoids the metastable persistence of high temperature phases that is a common problem in peritectic systems. Murphy [20] in his study of the silver-mercury system, for example, found that the gamma phase was readily formed by the diffusion of mercury into finely divided silver, but that it was completely suppressed by cooling from the liquid state. Because of the high rates of diffusion of mercury into tin reported by Prügel [21] among others, this method appeared particularly suited to the study of the mercury-tin system.

With these advantages in mind, a series of experiments was performed in which ingots of tin were exposed to liquid mercury for varying periods, annealed and then serially sectioned and analyzed. The exposure temperature and time and the annealing time were varied systematically.

### 6.1. Specimen Preparation

The individual specimens used for the diffusion studies were small cylinders approximately 0.65 inch in diameter and 0.40 inch in length. They were machined from induction-melted ingots, slightly larger in diameter and 3 to 4 inches in length, cast under vacuum in Pyrex. All of the ingots were prepared from the high-purity tin with the exception of a limited number of the initial ingots which were made from Baker and Adamson Reagent Grade Tin Sticks, of the composition given in table 2. Under the conditions of the test no differences in behavior could be detected between specimens made from this metal and from the higher purity tin.

The cast ingots were turned in a lathe to remove any surface imperfections and were then cut into cylinders approximately 0.40 inch in length. Any cylinders showing signs of piping or porosity were rejected, and the remainder were weighed and measured as a means of detecting gross internal porosity. If the specimens were stored before exposure to mercury, their surfaces were cleaned immediately before use by a light polishing on 600-grit silicon carbide metallographic paper.

### 6.2. Experimental Procedure

The specimens were exposed to mercury by immersion at constant temperature. In order to avoid excessive initial dissolution of the specimens, saturated solutions of tin in mercury were prepared at each diffusion temperature. Four nominal temperatures were employed, 37, 60, 85, and 110 °C. It was found possible to reduce the variation in temperature of the specimen itself to less than ±0.1 °C in all cases, by placing the beaker containing the immersed specimen within a vacuum desiccator, which was in turn placed within the oven.

After varying periods of immersion, the specimens were removed from the mercury and the excess liquid was blown from the surface with an air blast. This treatment did not remove all of the liquid, but did reduce the quantity to a thin film adhering to the surface. Some of the specimens were then sectioned immediately, while others were returned to the oven for an additional annealing period before sectioning.

**Figure 5:**
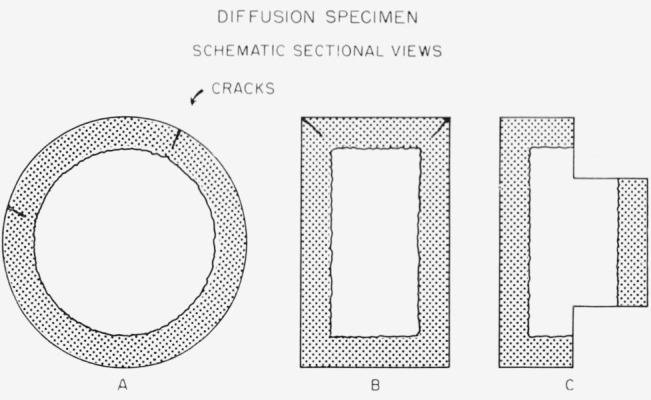
Schematic sectional views of diffusion specimen. Dark areas indicate diffusion of mercury.

All sectioning was done on a lathe. The specimen was held in a collet and the lateral surface turned down until unreacted tin was exposed. A series of samples was then taken from the mercury-containing layer remaining on the end of the specimen. Figure 5 shows a schematic representation of a specimen after immersion in mercury. Views (A) and (B) are transverse and axial sections and indicate mercury penetration as well as characteristic location of expansion cracks. View (C) is an axial section of a specimen as it would appear after the reduction of the lateral surface preparatory to the taking of samples as serial sections from the end. The depth of cut used in taking the samples varied from specimen to specimen, being adjusted so as to provide minimum sample of 80 mg. These samples were then stored at room temperature until analyzed for their mercury content.

Mercury analysis was performed by a modification of the technique of Crawford and Larson [22], employing an evacuated closed system rather than a carrier gas stream. The tube was evacuated to a total pressure of 5 to 10 mm of mercury and then flushed repeatedly with dry nitrogen before the valve was finally closed with the tube in the evacuated condition. The furnace was maintained at a temperature of 500 °C at the location of the combustion boats, and the samples were left in the furnace for 1½ hours. At the end of this period the Pyrex tube was slid from the furnace and allowed to cool to room temperature before the vacuum was relieved. The mercury distilled from the specimens condensed on the cool portion of the tube remaining outside of the furnace, and was removed mechanically before the combustion boats were withdrawn for reweighing. This procedure retained the advantage of the Crawford and Larson [22] technique in that the mercury content was determined as weight loss in the specimen rather than requiring the collection and determination of the mercury driven off. At the same time it minimized oxidation problems due to either leakage or trace contamination of the carrier gas.

Table 6 shows the conditions of test for all the specimens studied with the exception of calibration runs, specimens tested during the development of the methods, and some few specimens lost due to experimental error. The immersion and annealing temperatures were the same for all tin specimens annealed at 37 and 60 °C. Attempts to immerse a tin specimen for any extended period at either 85 or 110 °C led to the rapid conversion of the specimen and mercury to a slushy mass of platelike crystals dispersed in the remaining liquid. These specimens were therefore immersed at 37 °C prior to annealing at the higher temperature.

### 6.3. Results

**Figure 6:**
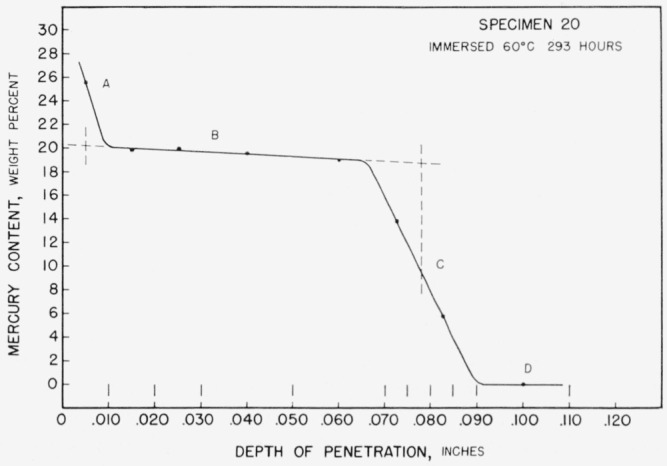
Concentration-depth curve for specimen 20.

The results of the analyses for specimen 20 are presented in figure 6. It exhibits many features common to all of the specimens and in particular of those sectioned immediately after removal from immersion. Each point on the diagram represents the result of an analysis of one entire sample and is plotted at the mean depth of the sample. The diameter of the points approximates the uncertainties of measurement of each value. The range of depth involved in each sample is indicated by the short bars near the lower margin of the figure.

The curve as drawn through the experimental points shows, as expected, a continual decrease of mercury content with depth. It also appears to consist of four distinct sections which are lettered A through D on the figure. Section A indicated a surface layer of high but rapidly decreasing mercury content. It is readily interpreted as a mixture of the equilibrium surface phase with the adherent mercury film. Section B indicates a thick layer of very slowly decreasing mercury content. Such steps in diffusion curves are commonly taken as indicating a one-phase layer. Section C, indicating a layer of rapidly decreasing mercury content probably represents a mixture of the phase of section B with that of section D. Section D, which here consists only of portions of zero mercury content, normally will include the unreacted core material and also the solid solution region of the same structure.

Considering the curve as a whole, it seems to show the presence of one intermediate tin-mercury phase. The composition limits of this phase may be estimated by extrapolation of the straight line of section B to the middepths of the transition zones. Such an extrapolation (as indicated by the dotted lines in fig. 6) leads to an estimated maximum mercury content of 20.3 percent and a minimum of 18.8 percent. A similar procedure can be followed for the estimation of the maximum solubility of mercury in tin, by extrapolation of the line in section D, in those cases where more than one nonzero point occurs in that section.

Caution must be exercised in reaching such conclusions, since several possibilities for error exist. It has been demonstrated radiographically by Gunther and Jehmlich [23] that the initial penetration of mercury into tin is intergranular; thus the possibility exists that the mercury content of the points in section B is too high because of the inclusion of such intergranular mercury. The possibility also exists that there might be one or more undetected solid phases in section C which remained undetected because a low diffusivity or narrow composition limits kept the layer thickness too small to be detected by the sectioning technique employed. Although the individual layers may be extremely thin, it is generally held that a separate layer must be formed for each intermediate phase. Rhines [24], for example, states that, “In binary systems, when diffusion occurs at substantially constant temperature and pressure, the layers formed correspond in kind and in order of their occurrence to the single-phase regions,… no two-phase regions appear.” If such phases exist undetected in section C of figure 6, then the proper limit for the extrapolation of the line of section B is to the midpoint of the transformation to the first such phase.

**Figure 7:**
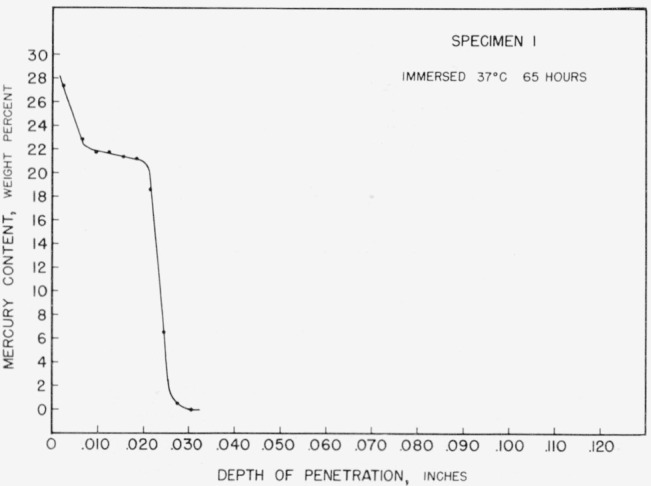
Concentration-depth curve for specimen 1.

The curves for all specimens sectioned immediately after removal from mercury at 37 or 60 °C were similar, each showing a single flat. In the specimens immersed at 37 °C, however, the extrapolated composition limits were approximately 22.3 and 21.0 percent mercury. Figure 7 shows the results for one such 37 °C specimen, specimen 1.

**Figure 8:**
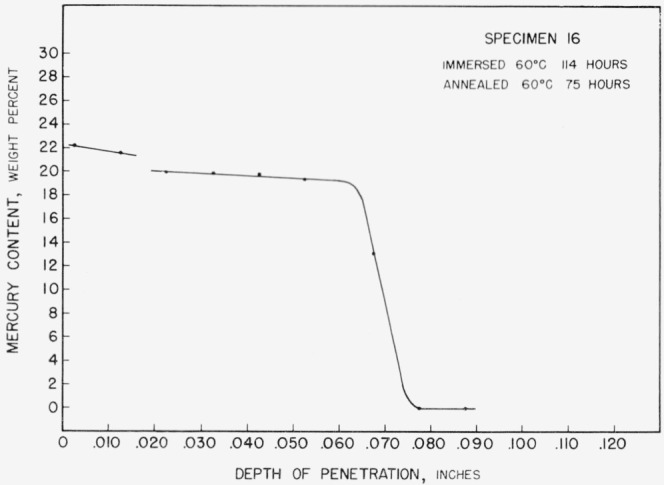
Concentration-depth curve for specimen 16.

One specimen, specimen 16, immersed and annealed for a short time at 60 °C, produced a diffusion curve showing two flats. As can be seen from figure 8 the composition limits for the two phases agree well with those found in figures 6 and 7

**Figure 9:**
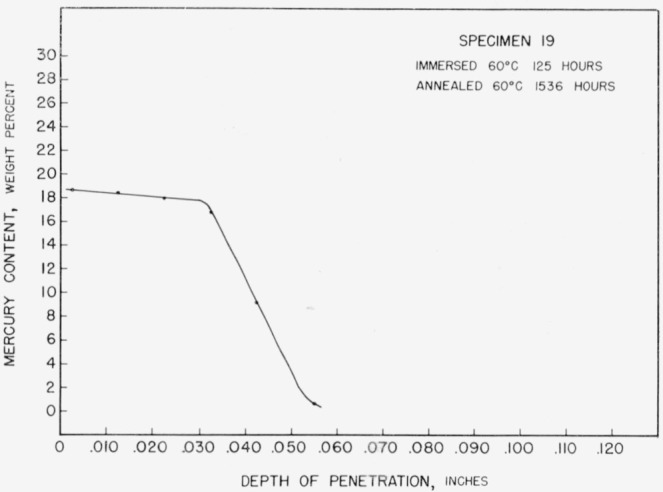
Concentration-depth curve for specimen 19.

**Figure 10:**
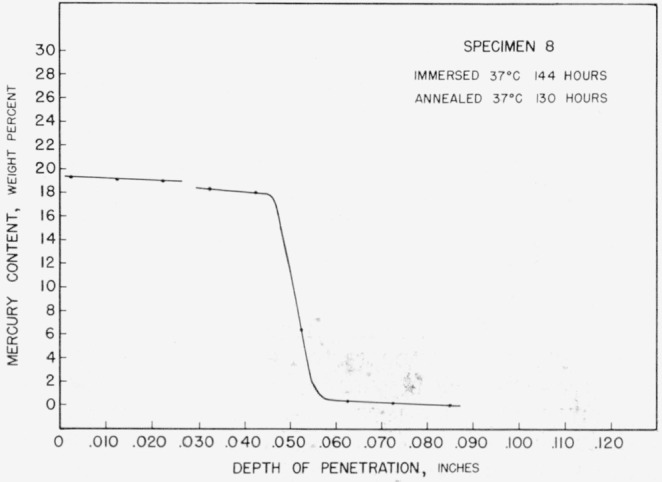
Concentration-depth curve for specimen 8.

Protracted annealing at either 60 or 37 °C resulted in the production of curves with a third set of indicated limits of mercury content, figure 9. The relatively short immersion time and long annealing time used for specimen 19 resulted in a complete transformation to the third observed phase. Slightly longer immersion and a short anneal produced steps characteristic of both the second and third phases in specimen 8, figure 10.

**Figure 11:**
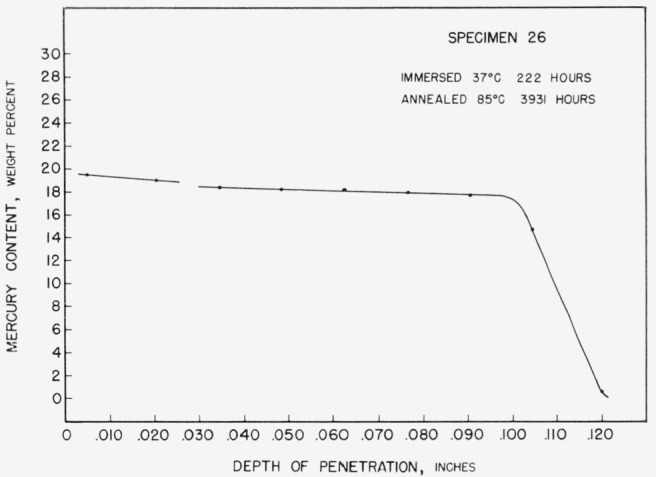
Concentration-depth curve for specimen 26.

Whereas specimens annealed at 37 °C would be “dry” within 24 hours, the specimens annealed at 85 °C showed liquid on their surfaces after 11 days. The specimens annealed at 110 °C showed persistent surface liquid up to 14 days. Even then the liquid mercury disappeared only after machining had exposed a fresh tin surface with which it could react. Further evidence of a low rate of diffusion at these temperatures is given in figure 11. When sectioned after 5½ months of annealing at 85 °C, specimen 26 still showed layers of the second and third mercury-containing phases.

Table 6 summarizes the results for all of the individual tin-mercury diffusion specimens. The composition limits for each observed flat were calculated by the extrapolation procedure used in figure 8 and are tabulated here in accordance with the presumed occurrence of three intermediate tin-mercury phases. Also included is the total depth to the point at which the mercury content is 10 percent. The composition limits reported in this table should be considered as saturated values only when the equilibrium phase was also present in the specimen.

### 6.4. Discussion

The method of constant temperature diffusion followed by serial sectioning and analysis appears to be well suited to the study of the tin-mercury system. The results indicate the formation of three intermediate tin-mercury phases at 37 °C.

The phase of lowest mercury content appears to correspond to the gamma phase of existing diagrams, since even protracted annealing does not cause the appearance of any phase intermediate in composition between it and tin. The phase with the next higher mercury content appears to correspond to the delta phase reported by Gayler [11] and decomposes at an appropriate temperature for such identification. The remaining phase is previously unreported but is here tentatively designated epsilon.

Table 7 presents the composition limits for these phases as determined at each experimental temperature. As shown in the table, these phases all have narrow and closely spaced zones of solid solubility. The most noteworthy finding is the unexpectedly high minimum content found for the gamma phase. This is in sharp contrast to previously reported limits for this phase which indicated minimum mercury contents of the order of 8 percent as seen in figure 1.

This previous solubility limit appears to be based primarily on the X-ray diffraction work of von Simson [9] and Stenbeck [8], with some indirect evidence being provided by the results of Løvold-Olsen [25], Schubert et al. [13], and Raynor and Lee [12]. It is very difficult in most of these instances to determine what was the previous thermal history of the specimens used. Almost certainly in the case of the work of both von Simson [9] and Stenbeck [8] and apparently in most of the other work, the annealing times were inadequate to cause the precipitation of tin from the gamma phase formed on cooling from the liquid. As a result, the composition limits of the gamma phase based on their findings are more indicative of the composition range over which the gamma phase is formed at elevated temperatures than of its equilibrium extent at room temperature. The one possible exception to the charge of insufficient annealing is a specimen of Raynor and Lee’s [12] which was annealed for 2 weeks at 150 °C. This specimen contained 7.193 atomic percent of mercury (approximately 11.5 weight percent), and apparently consisted entirely of gamma. While their paper does not deny the presence of tin lines in the X-ray pattern neither does it report them as it presumably would had they been observed. Possibly even this annealing time is inadequate or the amount of grain size of the precipitated tin was too small to detect. If not, a rather rapid widening or displacement of the gamma region must occur above 110 °C to accommodate this observation.

## 7. X-ray Diffraction

### 7.1. Specimen Preparation

X-ray diffraction patterns were obtained on a total of 20 mercury-tin specimens with mercury contents ranging from 5.0 to 22.1 percent. The specimens were prepared by two different techniques; 14 of them were derived from diffusion specimens while the remaining 6 were fused and annealed.

The technique employed in the preparation of the specimens by diffusion was identical with that by which the samples were obtained for mercury analysis. The samples used for the diffraction studies were selected, on the basis of the analyses of other samples from the same diffusion specimen, to provide evidence about the structures of the phases found in diffusion studies. The mercury content of these specimens was determined by interpolation of the composition-depth results from adjacent analyzed samples.

The remaining 6 specimens were prepared by sealing weighed portions of the component metals in a Pyrex tube in vacuum or an inert gas atmosphere. The alloy was fused at 250 °C, and quenched. The ingot was then annealed for a short period in the tube. After removal from the Pyrex tube the specimen was reduced to a coarse powder by turning on a lathe.

This procedure was intended to correspond approximately to the specimen preparation techniques used by previous investigators, although, as noted above, the information as to the exact techniques they used is often incomplete. The annealing times used in this study appear to exceed those of Stenbeck [8] and von Simson [9] but are less than those used by Raynor and Lee [12].

The composition and source of the X-ray diffraction specimens produced by diffusion are listed in table 8. The composition and heat treatment of the X-ray diffraction specimens produced by fusion are given in table 9.

### 7.2. Experimental Procedure

All of the diffraction patterns were obtained on a Norelco X-ray Spectrograph using copper K*α* radiation. This instrument is equipped with a goniometer having an auxiliary rotating device which rotates the specimen about an axis perpendicular to its surface throughout the test. This rotation produces more uniform curves when the specimen is small, as were the majority of those used in this study.

The specimens were prepared for the tests by sprinkling the particles onto a thin layer of petroleum jelly spread on the surface of the plastic mount. The diffraction curve was run over the range from 20° to 165° 2 *θ* at a rate of 1° 2 *θ* per minute, with a chart speed of ½ inch per minute. This scanning rate and chart speed were found to produce very satisfactory curves with good resolution. Even at 2 *θ* angles as low as 65°, the *α*_1_ and *α*_2_ peaks were normally resolved on the curves from specimens prepared by diffusion. At 2 *θ* angles above 110° they were often resolved to the background level. The resolution was somewhat poorer on cast specimens with the *α*_1_ and *α*_2_ lines routinely resolved only above 80° 2 *θ.*

### 7.3. Results

**Figure 12:**
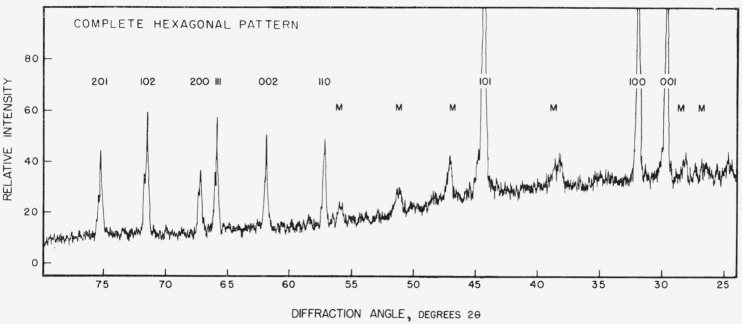
“Complete hexagonal” X-ray diffraction pattern.

The results of the X-ray diffraction tests on the specimens produced by diffusion correlate closely with the mercury content. Those specimens having mercury content between 21.0 and 22.2 percent mercury, that is specimens selected from the highest mercury content phase regions of diffusion specimens, uniformly produced curves of the type shown in figure 12. This curve was obtained from specimen number 3 and is readily indexed as a simple hexagonal structure with the parameters reported by Raynor and Lee [12] for the gamma mercury-tin phase. The individual peaks in figure 12 are labeled on this basis. (Lines labeled M are from the resin used as a specimen support.)

Although the diffraction curves were run from 20° to 165° 2 *θ*, only the portions between 25° and 80° are reproduced here. Most of the changes of interest occur within this interval.

All of the specimens taken from the phase with the lowest mercury content of the diffusion specimens (17.8 to 18.6% mercury) gave diffraction patterns similar to figure 12 except for the almost complete suppression of the 001, 002, and 003 peaks. Minor changes occur in the peak heights of other lines but those mentioned are the most characteristic. All of the lines which are present appear at the same angles as in figure 12.

Specimens having mercury contents between 18.6 and 21.0 percent gave patterns in which the ratios of the 001, 002, and 003 lines to the 100, 200, and 300 lines, respectively, increased approximately in proportion to mercury content.

**Figure 13:**
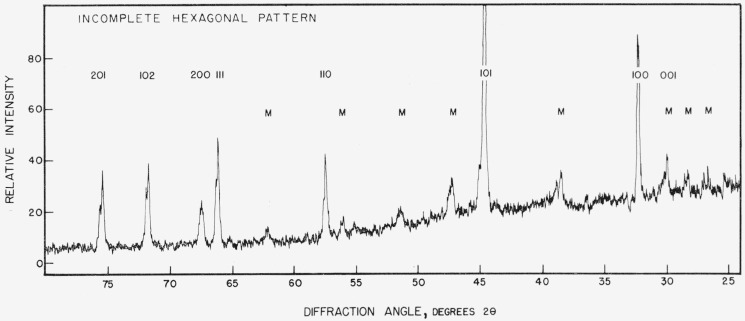
“Incomplete hexagonal” X-ray diffraction pattern.

Figure 13 shows a curve derived from specimen 10 which contained 18.72 percent mercury. Even this small amount of mercury in excess of the 18.6 percent limit has caused the reappearance of the 001 peak, although the 002 peak cannot be distinguished from the diffuse peak due to the support. More mercury increased the relative height of these peaks but in no instance did the 001 or 002 line of an intermediate mercury content phase specimen exceed 80 percent of the peak height of the 100 or 200 line. In contrast, in all of the highest mercury content phase specimens the 001 and 002 lines were stronger than the associated 100 and 200 lines.

Table 8 presents a summary of the X-ray diffraction findings on diffusion samples with the specimens arranged in order of mercury content. Only three specimens appear to merit further comment. Specimen 9 appears out of place. Its composition and source place it as an intermediate mercury content phase specimen, but its X-ray diffraction pattern showed 001, 002, and 003 lines among the strongest found. The cause of this conflict is not known.

The mercury content of specimen 6 similarly appears to be too low for the observed pattern. In this case, however, the mercury content is probably at fault. This specimen was the only one tested without sectioning, so that the pattern was obtained from the surface of the intact specimen rather than from a powdered layer. The mercury content was estimated from the composition-depth curve for the opposite end of the ingot.

On the basis of its composition and the shape of the diffusion curve, tin lines were expected in the pattern of specimen 11. They were not found. Inspection of the sample showed it to consist of two obviously different types of particles, one fine and granular, and gray in color; the other small, curled chips more nearly white in color. The mercury content of the mechanically separated chips was found to be about 4 percent. If this mercury were distributed as a thin surface layer of a tin-mercury phase, it might cover the tin and explain the absence of tin lines.

The X-ray diffraction patterns from the cast and annealed specimens showed a somewhat similar dependence upon mercury content as shown in table 9. In no instance was the pattern of the cast specimens of the type which has been called “complete hexagonal” in the diffusion specimens. It is believed that the short time at a low annealing temperature was inadequate to resolve a mixture of phases produced on cooling.

### 7.4. Discussion

The X-ray diffraction results appear to indicate the occurrence of two phases between 17.8 and 22.2 percent mercury in the mercury-tin system at normal room temperatures. These findings are in disagreement with previous X-ray investigations of this system and only partially corroborate the diffusion test results of this study. The nature of the observed patterns, however, are such that they may permit a reconciliation of the otherwise contradictory data.

The structure found for diffusion specimens with 21.0 to 22.2 percent mercury is readily indexed as a simple hexagonal structure with one atom per unit cell as reported by von Simson [9]. So also are most of the structures containing 18.7 to 21.0 percent mercury which were labeled “transition” structures. But the structure in equilibrium with tin at room temperature, the “incomplete hexagonal” in which the OOX lines are missing, cannot be explained with such a simple structure.

One possibility that must always be considered in regard to patterns in which particular lines appear to be suppressed is preferred orientation. The method of specimen preparation employed in these tests makes this an unlikely cause in this instance. The samples were reduced to a powder which was stored at room temperature for a considerable period of time before the patterns were taken. The sharpness of the lines in the patterns, showing no evidence of strain broadening, indicates that recrystallization probably occurred in the particles during this time. The specimens were prepared for the diffraction test by sprinkling the powder onto a layer of petroleum jelly spread on the surface of the mount. Even if preferred orientation did occur in the individual chips as a result of the machining operation, it seems unlikely that the particles could all be so alined after transfer to the mount. No alternate structure is proposed, but it is possible that some structure more complex than the one atom simple hexagonal is required to account for the suppression of the missing lines. *If* the patterns in figures 12 and 13 represent two different phases corresponding to the highest mercury content and lowest mercury content phases of the diffusion specimens, the “transition” pattern is easily explained as a mixture of the two. The X-ray diffraction results fail to confirm the existence of the intermediate content phase of the diffusion.

Although several specimens were tested with compositions in the immediate vicinity of that for which Stenbeck [11] reported a line doubling, which he attributed to an orthorhombic structure, no evidence of such a structure was found. Since the gamma phase is reported to extend to much lower mercury contents at elevated temperatures, it is conceivable that his results were the product of lattice parameter variations caused by variation in mercury content in unannealed specimens. He does not report any annealing treatment for his specimens.

The problem remains as to why other investigators have found the structure in equilibrium with tin to be a simple hexagonal if the 00X lines are truly absent. A possible explanation in the case of cast specimens is incomplete annealing. As seen in specimen 16, a composition (17.8%) which in a diffusion specimen would result in the complete absence of the 00X lines, does not do so in a cast specimen even after a short anneal. Cast specimens containing 8 to 14 percent mercury as von Simpson’s [9] did, might well produce at least some of the phase responsible for these lines and show a “transition” pattern. It is of interest to note that she reports the 001 line as weak and that she found the 002 line on only one side of the film.

Raynor and Lee [12] investigated a specimen with 7.193 atomic percent mercury (11.5 wt %) which was annealed for 2 weeks at 150 °C. They do not report the absence of the 00X lines or the occurrence of tin lines. This specimen lies between specimens 17 and 19 in composition and was annealed at approximately the same temperature for a considerably longer time. No previous investigator seems to have studied a specimen in the narrow range of 17.8 to 18.6 percent mercury. Even so, extended annealing would apparently be required to produce a uniform structure in cast specimens.

The X-ray results do little to substantiate the tin saturated boundary of the gamma region as inferred from the diffusion study. Only three specimens, 17, 18, and 19, have compositions and annealing temperatures that would make them of use for this purpose. The annealing times are certainly too short to assure equilibrium if allowance is made for the slow rate of diffusion found at elevated temperatures. (See fig. 11). The identification of weak tin lines in specimen 19 in spite of the very short anneal is, however, partial confirmation that the boundary does shift to higher mercury contents at lower temperatures. It is possible that specimens 17 and 18 fall within the gamma region and would not show tin lines even after extended annealing.

The failure to obtain tin lines from specimen 11 raises some doubt as to whether the method is suitable for the determination of this boundary. As mentioned before, the sample at the time of sectioning appeared to contain two distinct phases, one of which appeared to be tin. After standing at room temperature for some time before the X-ray pattern was determined, the specimen showed no tin lines even though chips of low mercury content were readily separated mechanically from the sample. This behavior can be explained if portions of the phase that was present with the tin has mercury contents higher than the equilibrium value as the result of a low rate of diffusion. After sectioning, these portions would be brought in direct contact with the tin chips and mercury transfer could occur, producing a layer of product on the surface of the tin chips and thus masking the tin lines. Such a mechanism could operate in any instance when equilibrium has not been attained prior to sectioning, but should at least in part be offset by determination of the diffraction pattern as soon as possible after sectioning.

If the gamma region is as curved as the diffusion results indicate, the appearance of tin lines in a diffraction pattern obtained at room temperature might be the result of precipitation from what was a homogenous structure at the annealing temperature. In a more general sense, the same possibility of transformation between the annealing temperature and the diffraction test temperature might be invoked to explain the appearance of only two phases where the diffusion tests indicate three. If provision were made for adequate annealing, elevated temperature diffraction tests would seem to offer the best hope of clarifying the boundaries of the gamma phase. Annealing times much longer than those of Schubert et al. [13] would be required.

## 8. Proposed Tin-Mercury Diagram

**Figure 14:**
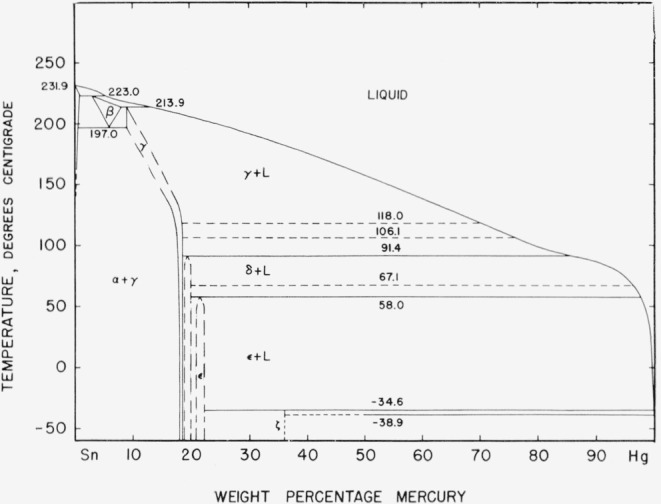
Proposed tin-mercury constitution diagram.

**Figure 15:**
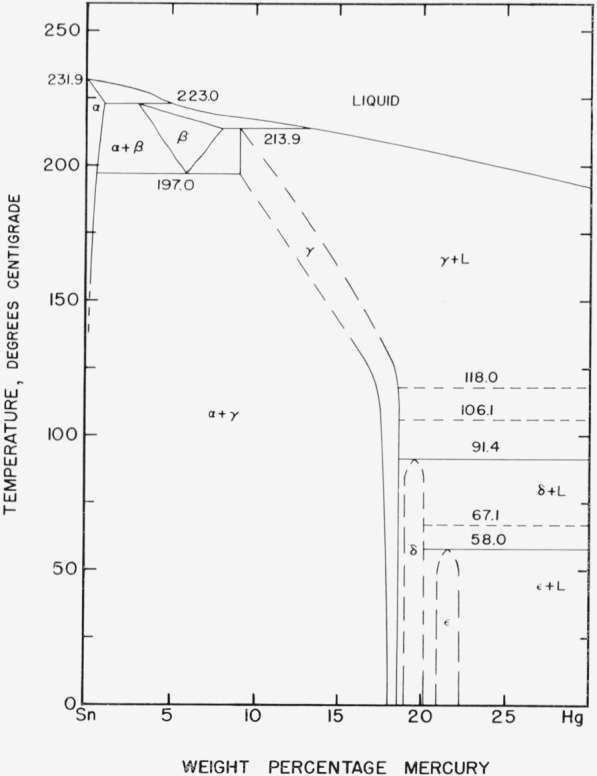
Enlarged tin-rich portion of proposed constitution diagram.

The proposed tin-mercury diagram based upon the findings of this study is shown in figure 14 and an enlargement of the tin rich end of the diagram is shown in figure 15. In drawing the boundaries in the diagram an attempt has been made to reconcile the results from various test methods and investigators. Where conflicts occur between the different sources, an attempt has been made to allow for the relative uncertainty of the individual findings.

The liquidus curve essentially follows that of van Heteren [6] except that it has been lowered slightly in the alpha+liquid and beta+liquid regions where he had no observations. This lowering is based on the thermal analysis results of this study and appears to agree with Prytherch’s [10] results in the same region. The alpha phase boundaries are based mainly on those as drawn by Hansen [3]. The maximum solubility of mercury in tin has been indicated to be 1 percent by van Heteren’s [6] electrode potential measurements, and the thermal analysis and metallographic results of this investigation confirm that this limit must be less than 2.0 percent mercury. There appear to be no other applicable data.

The existence of beta phase at elevated temperature appears to be well established. Our thermal analysis studies confirm Prytherch’s [10] finding of a peritectic arrest although the value obtained is slightly lower than that of figure 1. The only evidence against the occurrence of this phase was the work of Schubert et al. [13], which apparently was in error because of inadequate annealing of the specimens. In a note added in publication, they acknowledge that Raynor and Lee’s [12] results were conclusive. Although the existence of this structure is quite certain, the composition limits are unsupported by experimental data. As drawn in figures 14 and 15, they merely follow figure 1 for lack of any better information. The limits appear reasonable and are not contrary to theory.

The beta eutectoid temperature is very poorly established, although the thermal analysis results appear to confirm that the 198 °C value of figure 1 is approximately correct. If reliance is placed upon the presence or absence of experimental points in Prytherch’s [10] diagram, as noted in the comments on Gayler’s [11] paper, this temperature was never established experimentally. For lack of other evidence, it is indicated here at 197 °C on the basis of our thermal analysis results.

The gamma peritectic temperature of 213.9 °C also is based on thermal analysis results and agrees well with Hansen’s [3] value of 214 °C. The composition of the gamma peritectic is set at 9 percent mercury primarily on the basis of X-ray results of von Simson [9] and Stenbeck [8] although, since their results can be reconciled with the present findings only on the basis of incomplete annealing at low temperature, it is perhaps risky to assume that their specimens were at equilibrium above 200 °C. From the liquidus values it is obvious that this point must be at 14 percent mercury or less, but there is no evidence for a location other than the one indicated.

The remainder of the gamma region has been considerably altered. The tin-saturated boundary has been moved to agree with the results of the diffusion studies below 110 °C, as has the mercury-saturated boundary. Between 110 and 197 °C the tin-saturated boundary has been drawn to allow for the X-ray diffraction results on cast specimens. The mercury-saturated boundary above 110 °C has been drawn on the basis of heating curve indications of the start of melting. These data, however, showed considerable variation with annealing and the curve should be considered as approximate.

The overall picture of the gamma region as a narrow band swinging to higher mercury contents at lower temperatures is somewhat unusual because of the size of the swing relative to the width of the region. This construction serves nicely, however, to explain certain heat absorptions that occur in low mercury content thermal analysis specimens. In many instances the specimen has been annealed at an elevated temperature for a while before the start of the run. Portions of the specimen should thus have consisted of gamma saturated with tin. If the proposed diagram is correct, these portions would soon be heated across the gamma region and liquid would start to form with an absorption of heat. One example of this type of reaction is seen in figure 3.

Two additional peritectic phases labeled delta and epsilon are shown on the diagram. They are located on the basis of the combined thermal analysis and diffusion results. The delta phase was present in 85° diffusion specimens but not in 110 °C specimens. It is thus readily associated with the 91.4 °C arrest observed on thermal analysis. This thermal arrest is very strong and coincides with that found by Gayler [11].

The epsilon phase composition limits are similarly set from the observed composition range for the phase with the highest mercury content in diffusion specimens, and are even more certain than those of the delta region. The peritectic temperature is not so definitely known. The arrest found on thermal analysis at 67.1 °C is strong and well defined and seems certainly to represent a peritectic temperature. If this temperature is associated with the epsilon phase, however, highest mercury content phase layers should have routinely appeared in the 60 °C diffusion specimens but were detected in only one out of the eight specimens immersed at 60 °C.

The next lower arrest that might logically be associated with the highest mercury content phase structure occurred at 55.5 °C. This arrest is less well defined than the other, since it was found on heating curves only, and has an uncertainty of 2.5 °C. Considering that in a single instance this structure appeared in a specimen nominally annealed at 60 °C, the actual peritectic temperature would have to lie near the top of the uncertainty range even if maximum allowance is made for possible variation in the annealing temperature. As a result the peritectic temperature of the epsilon phase is indicated at 58 °C.

The peritectic phase at −34.6 °C found by van Heteren [6] and placed by Prytherch [10] at HgSn_3_ has not been investigated and is merely reproduced as previously stated. It is designated here as zeta.

This leaves three strong thermal arrests at 67.1, 106.1, and 118 °C to be explained. On the basis of the thermal data alone, these arrests would definitely appear to represent peritectic phase formations but no other affirmative evidence for such phases has been found. Such a closely spaced series of phases seems quite unlikely, and these arrests may instead represent second order transformations rather than phase changes. For these reasons these arrests are merely indicated by dashed lines in the figures.

One additional possibility, which is entirely speculative, is that the gamma region as shown in figure 15 is in reality two separate regions. One phase, stable at high temperatures, would form as indicated at the 213.9 °C peritectic temperature and would decompose on cooling at a 106.1 °C eutectoid. The other phase having the composition limits discovered in the diffusion tests would then be associated with a 118.0 °C peritectic temperature. Such a construction would account for two of the unidentified arrests and would simultaneously eliminate the need for the unusual variation in the composition limits of the gamma region with temperature. The tests as performed provide neither support nor refutation for such a construction. The findings of Günther and Jehmlich [23] seem to support the existence of some such complex series of phases although they do not identify any of the corresponding compositions.

## 9. Conclusions

The results of this investigation have indicated that the mercury-tin system is more complicated than was previously reported. Additional evidence for the existence of the beta phase has been found by determination of the separate peritectic temperatures of the beta and gamma phases as indicated by Prytherch [10]. The composition limits and eutectoid temperature of the beta phase remain to be confirmed. This appears best approached by a series of elevated temperature X-ray diffraction patterns. A set of specimens annealed in the beta range and tested at successively lower temperatures should provide the needed information. The data from thermal analysis studies and X-ray results suggest that the limits of the gamma phase should be shifted as indicated in figure 14 and 15. Corroborative evidence for Gayler’s [11] delta phase has been found by thermal analysis and diffusion methods. Possible evidence for an additional epsilon phase has also been found.

## Figures and Tables

**Table 1:** Composition of the mercury* used for the preparation of specimens

Element	Maximum content
	
	*ppm*
Combined noble metals (Ag, Au, Ir, Os, Pt, Pd)	1.0
Combined base metals	0.1
Mercury	Balance

* Refined mercury, Inorganic Chemistry Section, National Bureau of Standards.

**Table 2:** Composition of the tin* used for the preparation of initial diffusion specimens as reported by the manufacturer

Element	Maximum content
	
	*ppm*
As	3
Cu	20
Fe	100
Pb	100
Zn	100
Sn	Balance

* Baker and Adamson Reagent Grade Tin sticks, lot G303.

**Table 3:** Composition of the tin* used for the preparation of specimens as determined by spectroanalysis

Element	Maximum content
	
	*ppm*
Ag	1
As	**40
Bi	1.5
Cd	1
Co	1.5
Cu	15
Fe	2
Mg	0.5
Ni	3
Pb	15
Sb	10

* Tadanac Brand High Purity Tin Shot, lot HPM 522.

** Approximate value only; an error of 20 to 50% of the indicated value is likely.

**Table 4:** Nominal composition and liquidas temperatures of thermal analysis specimens

Mercury content		Specimen	Liquidus temperature a
		
*Weight %*	*Atom %*		° *C*
0.00	0.00	67	231.9
2.00	1.19	28	230.1
5.02	3.03	27	222.9
7.00	4.26	26	219.3
10.00	6.17	65	218.4
12.01	7.47	76	216.0
14.00	8.78	77	214.4
16.01	10.14	78	212.8
17.97	11.47	24	208.3
18.00	11.50	79	208.4
20.00	12.89	23	204.0
21.97	14.28	22	203.2
23.96	15.71	21	199.5
27.00	17.96	72	197.5
29.90	20.15	20	191.6
39.99	28.28	73	176.1
50.00	37.17	19	157.5
60.00	47.02	74	139.0
70.00	57.99	18	118.9
80.00	70.30	75	99.0
100.00	100.00	66	…………

a The estimated uncertainty of these values ranges from 0.1 to 0.8 °C, with an average of about 0.5 °C.

**Table 5:** Thermal analysis of mercury-tin alloys

Observed arrests temperature	Estimated uncertainty	Composition range	Identification and comments
			
*°C*	*°C*	*wt %* Hg	
231.9	0.02	0 to 5	Tin liquidus
223.0	.5	2 to 10	Beta peritectic
213.9	.5	2 to 20	Gamma peritectic
203.5	1.0	18 to 22	Structural artifact
197.0	1.5	2 to 10	Beta eutectoid
196.0	1.5	24 to 27	Unidentified
188.0	2.0	7 to 27	Unidentified
160.0	4.0	27 to 40	Unidentified
118.0	0.5	18 to 70	Phase change
106.1	.5	18 to 70	Phase change
91.4	.5	18 to 80	Gayler’s delta peritectic
67.1	2.0	30 to 70	Phase change—peritectic
55.5	2.5	18 to 70	Phase change—heating only

**Table 6:** Composition limits for the observed tin-mercury phases for individual diffusion specimens

Specimen	Conditions				Weight percent mercury content of three phases						Depth at 10% mercury
	Immersion		Annealing		Lowest		Intermediate		Highest		
	Temp	Time	Temp	Time	Min	Max	Min	Max	Min	Max	
											
	° *C*	*hr*	° *C*	*hr*	% Hg	% Hg	% Hg	*%* Hg	% Hg	% Hg	*in.*
1	37	65	…………	…………	…………	…………	…………	…………	21.10 (13.66)	22.30 (14.52)	0.023
2	37	72	…………	…………	…………	…………	…………	…………	21.00 (13.59)	23.00 (15.02)	.029
3	37	73	37	41	…………	…………	19.00 (12.19)	20.05 (12.92)	…………	…………	.037
4	37	73	37	611	…………	…………	19.00 (12.19)	20.50 (13.24)	…………	…………	.038
5	37	124	37	242	18.35 *(11.74)	18.50 (11.84)	19.15 (12.29)	19.50 (12.54)	…………	…………	.048
6	37	124	37	338	17.80 (11.36)	18.80 (12.05)	…………	…………	…………	…………	.049
7	37	144	…………	…………	…………	…………	19.20 (12.33)	20.20 (13.03)	21.50 (13.95)	22.05 (14.34)	.042
8	37	144	37	130	17.95 (11.46)	18.50 (11.84)	18.95 (12.15)	19.35 (12.43)	…………	…………	.050
9	37	165	…………	…………	…………	…………	…………	…………	21.30 (13.80)	22.60 (14.73)	.047
10	37	170	…………	…………	…………	…………	…………	…………	20.90 (13.52)	21.90 (14.23)	.046
11	37	264	…………	…………	…………	…………	…………	…………	20.80 (13.45)	22.20 (14.44)	.089
12	37	264	37	768	17.90 (11.43)	18.65 (11.94)	…………	…………	…………	…………	.089
13	37	336	…………	…………	…………	…………	…………	…………	20.90 (13.52)	22.20 (14.44)	.079
14	37	336	37	168	…………	…………	19.20 (12.33)	20.20 (13.03)	…………	…………	.077
15	37	366	…………	…………	…………	…………	…………	…………	20.80 (13.45)	22.00 (14.28)	.076
16	60	114	60	75	…………	…………	19.30 (12.40)	20.05 (12.92)	21.20 (13.73)	22.20 (14.44)	.070
17	60	114	60	510	17.80 (11.36)	18.70 (11.98)	18.95 (12.15)	19.00 (12.19)	…………	…………	.071
18	60	125	60	240	18.60 (11.91)	18.70 (11.98)	19.00 (12.19)	19.05 (12.23)	…………	…………	.054
19	60	125	60	1,536	17.75 (11.32)	18.65 (11.94)	…………	…………	…………	…………	.041
20	60	293	…………	…………	…………	…………	18.90 (12.12)	20.15 (12.99)	…………	…………	.077
21	60	293	60	721	17.80 (11.36)	18.65 (11.94)	…………	…………	…………	…………	.113
22	60	356	…………	…………	…………	…………	19.40 (12.47)	26.10 (17.29)	…………	…………	.100
23	60	356	60	606	17.75 (11.32)	18.55 (11.88)	18.90 (12.12)	20.05 (12.92)	…………	…………	.142
24	37	222	85	258	…………	…………	Samples lost		…………	…………	
25	37	222	85	335	…………	…………	19.00(12.19)	20.15 (12.99)		…………	.096
26	37	222	85	3,931	17.50 (11.15)	18.50 (11.84)	18.90 (12.12)	19.65 (12.64)	…………	…………	.110
27	37	222	110	332	…………	…………	Samples lost		…………	…………	
28	37	222	110	526	17.55 (11.19)	18.60 (11.91)	…………	…………	…………	…………	.092
29	37	222	110	672	…………	…………	Samples lost		…………	…………	
30	37	222	110	794	17.50 (11.15)	18.80 (12.05)	…………	…………	…………	…………	.096

* Equivalent atomic percentages.

**Table 7:** Composition limits for the gamma, delta, and epsilon tin-mercury phases as a function of diffusion temperature

Temperature	Mercury content					
	Gamma phase		Delta phase		Epsilon phase	
	Min	Max	Min	Max	Min	Max
						
*°C*	*wt %* Hg	*wt %* Hg	*ut %* Hg	*wt* % Hg	*wt %* Hg	*wt %* Hg
37	17.9 *(11.4)	18.8 (12.05)	19.0 (12.2)	20.1 (13.0)	20.9 (13.5)	22.3 (14.5)
60	17.8 (11.4)	18.6 (11.9)	19.0 (12.2)	20.1 (13.0)	**21.2 (13.7)	**22.2 (14.4)
85	17.5 (11.2)	18.5 (11.8)	18.9 (12.1)	20.1 (13.0)		
110	17.5 (11.2)	18.6 (11.9)				

* Equivalent atomic percentages.

** Found in only one specimen at 60 °C diffusion temperature.

**Table 8:** Observed X-ray diffraction patterns as a function of mercury content in diffusion specimens

Mercury content	Specimen	Source		Observed pattern
		Diffusion specimen	Sample depth	
				
*wt* %			*in.*	
22.10+ (14.37)*	1	7	0.0025	Complete hexagonal
21.94 (14.26)	7	1	.0075	Complete hexagonal
21.83 (14.18)	2	7	.0125	Complete hexagonal
21.72 (14.10)	3	11	.0250	Complete hexagonal
20.00 (12.89)	6	****15	Surface	Complete hexagonal
19.88 (12.80)	8	1	0.0375	Transition**
19.78 (12.73)	9	22	.0500	Complete hexagonal
19.27 (12.38)	12	26	.0135	Transition
19.20 (12.33)	4	8	.0075	Transition
18.72 (11.99)	10	21	.0500	Transition
18.29 (11.70)	13	26	.0555	Incomplete hexagonal***
18.19 (11.63)	5	8	.0375	Incomplete hexagonal
18.07 (11.54)	14	28	.0550	Incomplete hexagonal
12.58 (7.85)	11	21	.1075	Incomplete hexagonal

* Equivalent atomic percentages.

** Ratio of 001 and 100 peak height less than 0.8.

*** 001, 002, and 003 lines absent.

**** This specimen was annealed for 168 hours at 37 °C after removal from the mercury, in addition to the treatment indicated in table 6.

**Table 9:** Observed X-ray diffraction patterns as a function of mercury content in cast specimens

Mercury content	Specimen	Annealing		Observed pattern
		Temp	Time	
				
*wt %*		*°C*	*hr*	
21.75 (14.12)*	15	70	2	Transition**
17.83 (11.38)	16	70	2	Transition***
12.51 (7.80)	17	160	2	Incomplete hexagonal
12.45 (7.76)	18	160	4	Incomplete hexagonal
10.23 (6.32)	19	150	2	Incomplete hexagonal-tin trace
4.95 (2.99)	20	150	2	Incomplete hexagonal-tin

* Equivalent atomic percentages.

** Ratio of 001 to 100 peak height 0.75.

*** Ratio of 001 to 100 peak height 0.30.

## Footnotes

2 Figures in brackets indicate the literature references at the end of this paper.
